# The Bidirectional Engagement and Equity (BEE) Research Framework to Guide Community–Academic Partnerships: Developed From a Narrative Review and Diverse Stakeholder Perspectives

**DOI:** 10.1111/hex.14161

**Published:** 2024-08-01

**Authors:** Jennifer Cunningham‐Erves, Tilicia Mayo‐Gamble, LaNese Campbell, Bishop Calvin Barlow, Claudia Barajas, Jessica L. Jones, Karen Winkfield

**Affiliations:** ^1^ Division of Public Health Practice Meharry Medical College Nashville Tennessee USA; ^2^ Department of Health Policy Vanderbilt University Medical Center Nashville USA; ^3^ Department Community Health Behavior and Education Georgia Southern University Statesboro Georgia USA; ^4^ Second Missionary Baptist Cooperative Ministries Nashville Tennessee USA; ^5^ Vanderbilt Ingram Cancer Center Vanderbilt University Medical Center Nashville Tennessee USA; ^6^ Meharry Vanderbilt Alliance Nashville Tennessee USA; ^7^ Department of Radiation Oncology Vanderbilt University Medical Center Nashville Tennessee USA

**Keywords:** bidirectional, community–academic partnership, community‐based participatory research (CBPR), community‐engaged research (CeNR) framework, conceptual framework, equity

## Abstract

**Background:**

The engagement of community partners in equitable partnerships with academic teams is necessary to achieve health equity. However, there is no standardized approach to support bidirectional engagement among research stakeholders in the context of partnership equity at each phase of the research process.

**Objective:**

We describe the development of a systematic framework along with competencies and tools promoting bidirectional engagement and equity within community–academic partnerships at each phase of the research process.

**Design:**

We conducted a four‐step research process between November 2020 and December 2023 for framework development: (1) a narrative literature review; (2) expansion of existing bidirectional, equitable framework; (3) a scientific review with two groups of cognitive interviews (five community engagement researchers and five community leaders and members); and (4) three community‐based organization leader focus groups. Thematic analysis was used to analyse focus group data.

**Results:**

Using results of each step, the framework was iteratively developed, yielding four phases of the bidirectional engagement and equity (BEE) research framework: Relationship building and assessment of goals and resources (Phase I); form a community–academic partnership based on shared research interests (may include multilevel stakeholders) (Phase II); develop a research team comprising members from each partnering organization (Phase III); and implement the six‐step equitable research process (Phase IV). Bidirectional learning and partnership principles are at the core of the partnership, particularly in Phases II–IV. Competencies and tools for conducting an equitable, engaged research process were provided.

**Discussion:**

This conceptual framework offers a novel, stepwise approach and competencies for community–academic partners to successfully partner and conduct the research process equitably.

**Conclusion:**

The BEE research framework can be implemented to standardize the conduct of an equitable, engaged research process within a community–academic partnership, while improving knowledge and trust across partners and, ultimately, an increased return on investment and sustainability to benefit both partners in the area of health outcomes and ultimately health equity.

**Patient or Public Contribution:**

The development of this framework was co‐led with a community organization in which two leaders in the organization were equitably involved in each phase of the research process, including grant development, study design, participant recruitment, protocol development for focus groups and community and researcher review, framework design and content and dissemination of this manuscript as a co‐author. For grant development, the community leader completed the give‐get grid components for them as a partner. They also wrote up their lived experience in the research process for the progress report. For the focus groups, one community leader co‐led the focus group with the academic partner. For the narrative review, the community leaders did not actively conduct the narrative review but observed the process through the academic partners. One community leader wrote the section ‘relationship building’ and ‘bidirectional learning’ sections with the assistance of the academic partner, while they both equally provided input on other sections of the manuscript alongside academic partners. The community leaders have extensive experience in leading programmes, along with partnering with researchers to address health equity issues and improve health outcomes.

## 1 Introduction

Community–academic partnerships are necessary to advance health and improve outcomes [1, 2, 3, 4]. They are critical to developing and/or improving research, policy and interventions to achieve social justice in at‐risk communities [5, 6, 7]. Growing evidence suggests that these partnerships can facilitate high‐quality research and system changes necessary to translate research findings into action [8, 9, 10]. The application of community‐engaged research (CEnR) approaches is critical to ensure success in these partnerships and ultimately achieve equity and advance health [11]. Community‐based participatory research (CBPR), one approach to CEnR, is considered the gold standard [12]. It involves the active yet equitable involvement of community members, organizational representatives and researchers in all research phases to address social, structural and physical environmental inequities [13]. Each partner is an expert in their respective area, and their existing strengths can be leveraged to achieve health equity [2].

CEnR has various participation levels, ranging from researcher‐driven to community‐driven research approaches [12]. Historically, research initiatives were primarily driven by academic researchers, with the community's knowledge and ‘voice’ being lost in the process [14, 15]. There is increasing recognition of the value of engaging community members and organizations in the research process, including the increase in funding opportunities [12, 16, 17] [18, 19]. Additionally, there is a call to action for public health professionals to be trained in CBPR approaches [20, 21]. This paradigm shift calls for researchers, organizational representatives and communities to develop the competencies and capacity to conduct this work [1, 12, 17]. Roadmaps are needed to facilitate the development of equitable partnerships while ensuring all partners have the competencies needed to conduct the desired research tasks and ultimately promote health equity.

### 1.1 Existing Frameworks

CBPR principles exist to promote partnership equity and social change. Studies have explored equity in community–academic partnerships and established programmes promoting equity in these partnerships [3, 22, 23, 24, 25, 26]. Furthermore, studies have identified methods and measures to determine partnership synergy [4, 27, 28, 29, 30, 31, 32, 33]. A paucity of studies has established a framework to guide equity in community–academic partnerships [34, 35]. Yet, there is limited guidance on the steps (‘The How’) for bidirectional yet equitable engagement at each phase of the research process with a goal to advance health outcomes and equity [36]. To advance the field, a framework is needed that: (a) expands our understanding of processes and methods that ensure equitable engagement of academic and community partners in the research process; (b) helps us to understand the importance of equitable engagement in research from academic and community perspectives and associated competencies; and (c) demonstrates the potential impact on knowledge, attitudes, subjective norms and willingness, and participation related to prevention behaviours (e.g., research participation).

### 1.2 Preliminary Work

Meharry Medical College (co‐author J.C.‐E.) and Vanderbilt Ingram Cancer Center (co‐author C.B.) partnered with Second Missionary Baptist Cooperative Ministries (SMBC) (co‐authors B.C.B. and L.C.) in 2018 to develop, implement and evaluate the African American Men Cancer Summit ‘Healthy Men, Healthy Communities’. This is a 2.5‐h, culturally informed programme to educate African American men about cancer. The full programme is described here [37]. Members of the partnership (co‐authors J.C.‐E., B.C.B. and L.C.) identified and consolidated literature representing models, frameworks and strategies related to CBPR, CEnR and partnership equity to establish a framework to be implemented by community–academic partnerships to guide the conduct of research processes using bidirectional, equitable engagement approaches (File S1). This partnership later expanded to include health equity and community engagement experts to provide their knowledge based on experiences (co‐authors T.M.‐G. and K.W.) and input on framework refinement processes (coauthor T.M.‐G.) to ensure comprehensiveness.

### 1.3 Purpose

We describe the development of a practical framework using a stepwise, stakeholder‐guided approach that promotes bidirectional engagement yet equity in community–academic partnerships conducting the research process. We also offer competencies and strategies to ensure bidirectional learning between each partner conducting the research process while improving research competency, capacity and implementation.

## 2 Materials and Methods

**Figure 1:**
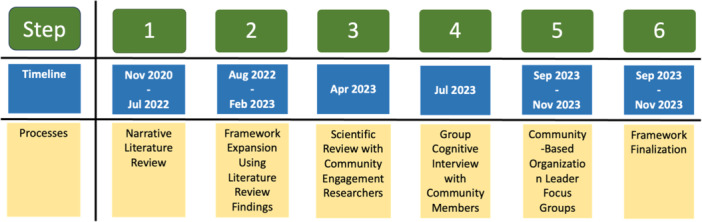
Framework development process.

We used a multi‐stage approach to develop a framework to promote equitable, bidirectional engagement in the research process (Figure 1). All procedures were approved by Meharry Medical College IRB (Protocol #: 23‐05‐1305) and expedited with written informed consent procedures. Study reporting followed COREQ guidelines [38].

### 2.1 Step 1: Narrative Literature Review, November 2020–July 2022

To gather concepts for this framework, we conducted a narrative literature review using a ‘purposive’ search and then selected articles. This review protocol prepared was not registered. The review was conducted in three electronic databases *PsycINFO*, *PubMed* and *CINAHL* from November 2020 to July 2022. Search criteria used key terms ‘framework’, ‘model’, ‘community–academic partnerships’, ‘bidirectional learning’, ‘health equity’ and ‘CBPR’ across the databases. These search teams were centred around three concepts: frameworks and models related to community–academic partnerships, partnership and equity principles, and health equity via community engagement. Models and principles related to community–academic partnerships, along with research and partnership equity were selected (File S2).

### 2.2 Step 2: Framework Expansion, August 2022–February 2023

We identified key concepts for a bidirectional, equitable framework of engagement using literature search findings. Using a deductive–inductive approach, we coded the articles and linked the codes. The expanded framework depicts a process and partnership principles for bidirectional engagement of community–academic partnerships to conduct research. We mapped concepts reflecting the conduct of those steps equitably by both partners. Last, we identified tools and resources to enhance each partner's knowledge and competencies to promote equity in conducting the research process.

### 2.3 Step 3: Scientific Review With Community Engagement Researchers, April 2023

We identified five research experts to reflect on the meaning of and strategies to achieve and sustain bidirectional engagement and equity within community–academic partnerships conducting research. These experts were also presented with the framework to modify while generating new framework content, and ensuring comprehension, accuracy and relevance. The selection criteria were: (1) having prior experience in conducting research through a community–academic partnership; and (2) willingness and ability to complete the review. A trained researcher in qualitative research methodology used brainstorming, a group decision‐making method, to obtain direct verbal feedback. Before the 90‐min group review, participants took a brief, demographic survey, and then the researcher led the review. For the framework specifically, the facilitator provided an overview and allowed 5 min for each expert to individually reflect for discussion with the other experts. Each phase of the framework was discussed with the researchers. After data were transcribed, two trained qualitative researchers coded the text using a priori codes ‘keep’, ‘remove’, ‘add’ or ‘clarify’ in a line‐by‐line coding technique (Example 1: ‘I like the partnership principles in the framework. Now that is a powerful component in this framework.’ [Code: Keep]; Example 2: ‘This framework does not show the relationship‐building phase which is needed to create an equitable community–academic partnership.’ [Code: Add]; Example 3: ‘I do not believe that bidirectional learning will occur throughout the research process. This will really depend on the degree of engagement of the partners.’ [Code: Clarify]). Discrepancies were discussed and resolved. Constant comparative analysis was used to compare codes iteratively, determining steps and processes to be added, removed, kept or clarified. Participants were compensated with a $50 gift card.

### 2.4 Step 4: Community Consultation, July 2023

We further explored bidirectional yet equitable community–academic partnerships and how the proposed framework displays those views from two community members and three community advocates in a 45‐min community consultation [39]. The selection criteria were as follows: (1) at least 18 years old; and (2) interested in/had prior experience in conducting research through a community–academic partnership. Data collection and analysis were completed as described in Step 3. Participants were compensated with a $30 gift card.

### 2.5 Step 5: CBO Leader Focus Groups, September–November 2023

#### 2.5.1 Study Design and Setting

We conducted three small focus groups (FGs) (total: *n* = 13; FG1: *n* = 4; FG2: *n* = 4; FG3: *n* = 5) among leaders of CBOs in Nashville, Tennessee. We applied a qualitative, descriptive study design [40] to gain a straightforward explanation of perception and experiences related to bidirectional engagement and equity for community–academic partners conducting research along with associated facilitators and barriers and perceived strategies for improvement. They also provided input on the framework for comprehension, accuracy, and relevance. Three FGs were chosen as three to six FGs are needed to meet coding [41] with a minimum of four participants per group recommended [42].

#### 2.5.2 Sample and Recruitment

A purposive sampling method was used to identify leaders of CBOs. Inclusion criteria were as follows: (1) female or male; (2) English‐speaking; (3) age 18 and older; and (4) had interest in or prior experience in a community–academic partnership as a CBO lead. Participants were recruited through the Meharry Community Engagement Core volunteer database and word of mouth.

#### 2.5.3 Data Collection and Analysis

A trained qualitative researcher (co‐author J.C.‐E.) facilitated using a 6‐item moderators' guide and another (co‐author T.M.‐G.) took notes for the 60‐min FGs. Participants provided consent and completed a brief, sociodemographic survey via REDCap [43]. FGs were audio‐recorded and transcribed verbatim. Compensation was a $40 gift card. Two trained, qualitative researchers (co‐authors J.C.‐E. and T.M.‐G.) with social science backgrounds analysed the transcripts in Excel version 16.79.1. A thematic approach [44] was used to analyse the data. Each transcript was read and notes were taken by each individual researcher to combine with the literature to develop a coding frame for further analysis. Each coder analysed the transcripts, organizing and categorizing the data to add to the coding frame. All discrepancies identified were resolved. Using a constant comparison method, codes were viewed for patterns and emerging themes identified.

### 2.6 Step 6: Framework Finalization, September–November 2023

We finalized the framework's structure and content using: (1) the literature; (2) CBO leader FG findings; (3) feedback from researchers and community members; and (4) our experiences documented at our weekly team meetings. A subgroup of the research team (J.C.‐E., T.M.‐G. and L.C.) discussed recommendations after each step and iteratively updated the framework's phases, steps and processes. These multiple data sources were triangulated to establish rigour. All team members reviewed and approved the final framework.

## 3 Results

### 3.1 Step 3: Scientific Review

Researchers perceived that CBPR principles offered some guidance or they did not offer enough information on how to engage with community partners equitably. Yet, all viewed community–academic partnerships should be equitable and highlighted strategies for equity (e.g., addressing power, ‘circular learning’, determining equity in the research process). Barriers to creating equitable partnerships included structural racism and classism, institutional pressures or glass ceilings (e.g., IRBs not accepting community partners in research roles) or community partners perceiving researchers should create equity. Facilitators to creating equitable partnerships were both partners participating in training and continued advocacy for community engagement in different research capacities. Using this information and framework suggestions, external factors, needs and capacity assessments and partnership and implementation outcomes were added to the framework.

### 3.2 Step 4: Community Consultation

All participants were African American and female. Community participants' description of an equitable partnership' included partners understanding each other's needs for research, co‐selecting and implementing a research strategy and outcomes with co‐ownership and identifying each partner's roles and responsibilities. Barriers to equitable partnership were their fear and perceived outcomes of the research process, continued lack of inclusion in the research process and the next steps and the onus being placed on the community to address health inequities with a negative description of their communities in research findings. Facilitators were relationship building, transparency and honest information exchanges, equitable engagement in the research process and ongoing relationships with the community from academic institutions and researchers. Using this information and framework suggestions, inputs (e.g., time) as a part of relationship building were added to the framework.

### 3.3 Step 5: CBO Leader FGs

All participants had a college degree. The majority were married (63.4%), female (76.9%) and had health insurance (69.3%). Over one‐third (38.5%) of participants' household income was less than $80,000. The mean age was 49.5 years. A majority (61.5%) had participated in research and about one‐third (30.8%) stated their organization partnered in studies. Of those four CBO leaders, one found the partnership very equitable (Table 1). Four themes emerged from FGs.

**Table 1:** Sociodemographics for African American CBO leaders (*n* = 13).

Education (*n*, %)	
Some college	2 (15.4)
Associate's degree	2 (15.4)
Bachelor's degree	3 (23.1)
Master's degree	5 (38.5)
Doctoral degree/professional degree	1 (7.6)
Age (mean, SD)	49.5 (0.69)
Marital status (*n*, %)	
Single	2 (18.2)
Married	7 (63.4)
Divorced	4 (18.4)
Gender (*n*, %)	
Male	2 (18.2)
Female	10 (76.9)
Other	1 (4.9)
Insurance (*n*, %)	
Tenn care	3 (23.1)
Health insurance through employment	3 (23.1)
Private health insurance	3 (23.1)
None	2 (15.4)
Other	2 (15.3)
Household income (*n*, %)	
$40,000 or less	3 (23.1)
$40,001–80,000	2 (15.4)
Over $80,000	6 (46.1)
Prefer not to say	2 (15.4)
Ever participate in research (*n*, %)	
No	5 (38.5)
Yes	8 (61.5)
Organization ever partner for research studies (*n*, %)	
No	5 (38.4)
Yes	4 (30.8)
I don't know	4 (30.8)
If yes, was the partnership equitable? (*n*, %)	
Not at all equitable/slightly equitable/somewhat equitable	3 (75.0)
Very equitable/extremely equitable	1 (25.0)

*Note:* All categories may not equal 13.

Theme 1 Equitable partnerships with bidirectional learning are necessary and a priority.

All CBO leaders viewed equitable partnerships with bidirectional learning as necessary and a priority to address health inequities. Others defined *equitable*, with one participant stating, ‘all decision‐makers in research having an equal say and vote in the research proposed’. Other views included working with diverse CBO leaders and communities in partnerships and negotiating study intent and design along with the roles of each partner during grant writing phases. This was deemed critical as each partner needed each other due to the resources, knowledge and experience each offered.

Theme 2 Multilevel barriers impede equitable community–academic partnerships.

#### 3.3.1 Societal/Community/Organizational Level

The impact of structural racism on establishing equitable and sustainable community–academic partnerships was emphasized by CBO leaders. One participant stated, ‘I think previously in the roles that we play in our research, it is very clear where we are on this side of research. We are the ones that are training the doulas and putting them out into the community. So, we are analysing whether or not the information we are giving them in the training is sufficient and we are analysing whether or not our clients are, or if we are seeing better survival rates among families of colour.’ A few highlighted the inequities in social determinants of health due to inequities in national policies that influence community/organizational level policies. Others discussed how IRB policies make it difficult or deny community partners in research leadership roles.

#### 3.3.2 Interpersonal Level

Barriers at the interpersonal level, the most commonly cited, were related to the researcher and the process. Researchers were perceived not to be ‘humane’, having limited cultural sensitivity, lacking skills, not listening or fearing to engage with the community and its leaders, which could alter a ‘researcher‐driven yet beneficial agenda’. Another presented post‐award grant issues (e.g., selection of incorrect community partner, unsuccessful recruitment procedures, poor resource distribution) due to little‐to‐no community input. ‘How are we supposed to be a team, but you do not understand what I do? … There are no mathematical solutions for disparities to go away’, a CBO lead stated.

Barriers related to the research process were being asked the same and/or incorrect research questions by different researchers with no change in actual issues discussed in the research study. A few identified competition among researchers and among CBOs, making it hard to find sustainable solutions. Many described researchers as unwilling or unwilling to offer to train CBO leaders for different roles in the research process. One CBO lead stated, ‘engaging in partnerships without proper training can potentially cause more harm than good’, and training will ‘help me to say yes to help you’. Another explained, ‘doing it for us, we never learn’.

Other barriers mentioned infrequently were the perception that researchers found CBO leaders intimidating or bullying and potentially discriminating against community partners. Researchers' inability to provide support (i.e., manpower, funding) when ‘life’ happens in the community, along with lack of access to researchers for opportunities and race‐concordant community–academic partnerships was stated and affirmed.

#### 3.3.3 Individual Level

Almost all CBO leaders acknowledged they may not have the capacity, resources, knowledge or skills necessary to engage in an equitable partnership. A few even highlighted the existence of language and research literacy barriers.

Theme 3 Strategies and tools to facilitate bidirectional, equitable partnerships.

#### 3.3.4 Strategies

CBO leaders stated that researchers should learn from community partners and other researchers with a lack of micromanagement. ‘There are too many chiefs and not enough Indians’, one CBO lead stated. Before the initial meeting of partners, researchers should provide documentation (e.g., card, pamphlet, one‐pager) to inform the CBO lead and allow preparation. All leaders highlighted that it takes time to build relationships to later lead into partnerships with one participant stating it took a minimum of 3 months to form a relationship. A few even stated that the setting for engagement is critical to ensure both partners are comfortable and feel ‘safe so that the lived experiences of both partners are not lost’. Trainings should be provided when needed to conduct research roles. Communication before, during and after research is essential.

#### 3.3.5 Tools

Meet‐and‐greets, coffee dates and databases of community partners and researchers were identified for relationship building. A point of contact at academic institutions should be identified so that CBO leaders will trust the request from the researcher(s). Speed dating sessions were suggested to determine partnership potential and parameters. The give‐get grid and workshops were highlighted to establish equity in the partnership.

Theme 4 The BEE Research Framework is a roadmap to directional, equitable community–academic partnerships, but improvement is needed.

Overall CBO leaders found the framework an acceptable representation of how equitable, bidirectional partnerships should be formed and carried out in the research process. Terms such as ‘co‐creating research’, ‘interactive’ and ‘work together’ were used to describe the framework. One CBO leader stated, ‘It's like you came into our mind while we were [sleep] to produce this.’ For relationship building, CBO leaders stated it was important to remove the ‘alphabet soup from behind the name’ and ‘disrobe everyone’ to set the tone. For input, both partners should give training opportunities on each other's processes and skills. Also, they perceived resources offered by researchers should close the gap for their participation as a community partner. Framework improvements included demonstrating where community members can be engaged, defining terms to ensure understanding of framework language and highlighting the return on investment for partnership participation.

### 3.4 Final Framework

**Figure 2:**
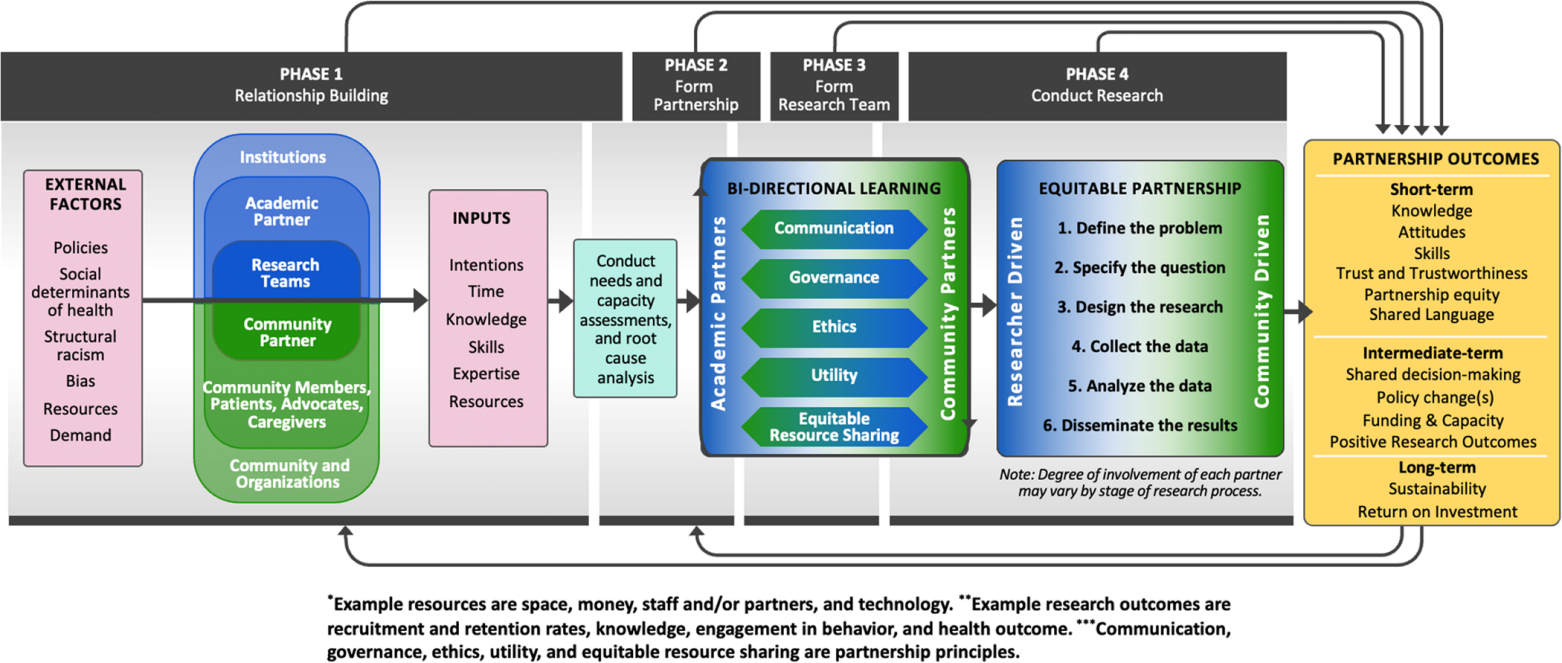
The bidirectional equity and engagement (BEE) research framework.

The BEE research framework is a four‐phase process for academic and community partners to conduct a six‐step research process using bidirectional, equitable engagement processes (Figure 2). The framework offers a systematic process of relationship building, partnership development and each partner's varying degree of involvement in each stage of the research process. Bidirectional learning is supported within and outside of the partnership, and outcomes of engaging in these partnerships are defined. A description of each phase is described below. The steps in each phase along with the competencies and tools of both academic and community partners are in Table 2. Table 3 shows the partnership principles that can serve as process evaluation measures.

**Table 2:** Competencies, tools and resources to conduct the BEE research framework.

Steps	Competencies	Tools and/or resources
Phase 1: Relationship building		
1. Explore potential partners 2. Identify shared health priorities (if any) 3. Explore research interests 4. Initiate a needs assessment 5. Initiate a capacity or readiness assessment	Academic partner Receive training on community engagement and community‐based participatory research Communicate policies and culture of the academic environment Acknowledge past research abuses when needed Go to community events (e.g., health fairs, churches, schools, parties) Implement trust‐building strategies Verbalize research interests and parameters of research Facilitate bidirectional learning Define a needs and capacity assessments and/or root cause analysis Community partner Communicate policies and culture of the organization and community Attend programmes or research opportunities (e.g., research team meetings, workshops and trainings, conferences) Verbalize community needs and explain *what has* and *has not worked* and *why* Re‐enforce bidirectional learning and importance of being trustworthy	Principles of community engagement AAMC principles of trustworthiness Diversity, equity and inclusion principles (e.g., use of plain language, disability accommodations) Community asset mapping Community engagement training for researchers Antiracism training and resources Speed dating Meet‐N‐Greet CommunityToolBox Capacity and needs assessment tools (e.g., logic model, surveys, interview guides) Root‐cause analysis Positionality map Problem tree
Phase 2: Form partnership		
1. Confirm partners and potential stakeholders 2. Establish rules of engagement 3. Select research project and determine outcomes 4. Develop grant proposal (optional)	Both partners Develop memorandum of understanding Develop a common language Establish governance, roles, responsibilities and time commitment Determine each partners' degree of involvement in each research phase Determine resources and sharing plan Promote shared discussion and decision‐making Be culturally sensitive Understand needs and capacity assessment data Identify research opportunities and select one Identify grant mechanism(s) Develop a grant proposal that demonstrates equity in roles, responsibilities and budget Obtain IRB approval (if the grant is funded and/or research is conducted)	Partnership principles Diversity, equity and inclusion principles (e.g., use of plain language, disability accommodations) Good clinical practice Belmont principles CBPR principles Give‐get grid Memorandum of understanding Capacity and needs assessment tools (e.g., logic model, surveys) SWOT analysis Concept mapping Delphi methods Nominal group process Shared language and dictionary Legal counsel (if needed) Regulatory/IRB consultation
Phase 3. Form research team		
1. Select team members from academic institutions and CBO 2. Identify community members to serve as advisors or consultants 3. Set times to meet with the team and each individual	Academic partner Conduct mock research project reviews Both partners Determine if the current role of each individual allows the opportunity to participate as a research team member Review interests, skills and time availability of potential team members Determine roles on the research team and who will manage the team Determine the degree of involvement of each research team member at each phase of the research process Identify training opportunities to support the roles of each team member	Team building activities Personality tests Regulatory/IRB consultation
Phase 4. Conduct research process		
1. Define the problem A. Discuss needs based on experiences B. Review literature and existing documents	Academic partner Teach community partners on how to conduct a literature search, needs assessment and capacity assessment Community Explain the needs of the community Identify factors influencing the health outcomes of the community Both partners Discuss potential strategies to address needs	Needs sssessment Capacity sssessment Scientific databases (e.g., PubMed, CINAHL) Crowdsourcing Community coalition
2. Specify the question A. Develop a research question B. Refine the research question	Academic partner Define research question Discuss components of the research question Demonstrate good and bad research questions Community Assist in identifying components of the research question Identify ways to increase comprehension of the question Both partners Co‐define the research question using literature and experiences	PICOT framework FINER criteria PEO framework
3. Design the research	Academic partner Train community partners on research methods, interventions (if needed), study designs, sampling and recruitment processes Ensure community partner(s) can differentiate between quantitative, qualitative and mixed methods Explain how target audiences are defined in research Community partner Explain the rationale for choosing or not choosing a target audience(s) Describe why a study design and/or recruitment strategy would or would not work for the target audience Offer new recruitment strategies Both partners Select study design and strategies for recruitment and data collection	Research methods training programmes for community members
4. Collect the data	Academic partner Train community partner on the data collection process including delivery of instructions, informed consent process and data entry and storage Community partner Educate academic partner on data collection strategies Both partners Inform potential questions participants may ask to prepare answers that are accurate and culturally sensitive	Survey data collection tools (e.g., Redcap, SurveyMonkey, Qualtrics)
5. Analyse the data A. Select software B. Clean data C. Review hypotheses D. Select statistical test(s) E. Analyse data	Academic partner Train community partner on basic statistical analyses and data interpretation Train community partner on qualitative data analysis and interpretation Train computer partner on how to prepare (e.g., entry, transcription) and manage (e.g., coding) data Community partner Provide researcher with potential questions to explore with the data Inform strategies to present the data	Quantitative Software (e.g., SPSS, R, SAS, Excel) Qualitative Software (e.g., Nvivo, Dedoose, Atlas.ti, Excel)
6. Disseminate the results A. Develop, submit and publish manuscript(s) B. Develop and submit abstracts and proposals for conferences C. Develop and disseminate findings to the community	Academic partner Offer or identify training in manuscript writing, public speaking, data presentation and PowerPoint development Explain guidelines and ethics related to authorship Explain how to use a reference software system Identify conferences for community members to attend Build competency of community members to develop abstracts, and present oral and poster presentations Develop competency for community members to develop and implement dissemination products Listen to community partners on the best strategies to disseminate research findings to the community Community partner Inform dissemination strategies and content for the community Develop products for dissemination to the community Disseminate products through the community Both partners Identify authorship order on papers and posters Determine roles in manuscript writing Determine roles in oral and poster presentation development	Authorship grid Community‐engaged research Dissemination model Pass‐it‐on Training for researchers on community‐engaged research dissemination Reference software (e.g., EndNote, Zotero)

**Table 3:** Partnership principles.

Principle 1. Ethics
Ethics refers to conducting the research process in a way that is acceptable and protects the welfare of the community. This includes respect for persons, beneficence and justice. Academic researchers should be trained and uphold those principles. When engaging community members as equitable partners in research, it is also important for them to be knowledgeable on the ethical conduct of research, including the Belmont Principles and the Declaration of Helsinki. Both academic partners and community members should receive human subjects training before conducting a research study.
Principle 2. Utility
A goal of mutually beneficial community–academic partnerships for research is to engage in activities that result in a benefit for each partner. Utility ensures the research activities meet the needs of both partners. Therefore, the contributions and benefits to each partner should be included in writing in the early stages, which establishes expectations throughout the project duration. This may also reduce the likelihood of conflict and potentially maximize the usefulness of the partnership and project outcomes for each partner.
Principle 3. Communication
Bidirectional communication is essential when academic and community partners are partnering to conduct the research process. The process is the exchange of information and the use of inclusive language among community and academic partners. It involves open, honest and respectful dialogue to build trust and credibility. The dialogue should be comprehendible, accurate and culturally sensitive. Throughout the research process, both partners should inform and update each other through scheduled check‐ins on the changes that occur in the process, capacity and expectations. Last, partners should always ask questions when there is a lack of clarity on roles, responsibilities and process. It is important to remember that training or coaching can be offered to improve communication among the partners.
Conflict resolution: A major component of bi‐directionalbidirectional communication is the ability to apply conflict resolution skills. During the research process, there can be differences in opinion on the proposed objective, methods and outcomes for the proposed study. Being able to be respectful, professional, listen, transparent and collaborate are some key attributes to preserving the partnership when conflict arises.
Principle 4. Shared Governance
Shared governance allows all partners to be effectively engaged and involved in decision‐making. Establishing the process of shared governance requires effective leadership, a multidisciplinary team and respect for each partner's structure and culture. The guidelines for shared governance should be established before research planning and implementation. However, shared governance is an ongoing and fluid process that requires continual assessment and flexibility to be responsive to each partner's and target population's needs. Before the study initiative, a MOU and an outline of roles and responsibilities is necessary to establish ground rules. During this time, discussions should facilitate an understanding of each partner's background, needs and constituency. This process also facilitates transparency between partners and potentially reduces the likelihood of conflict. Issues of transparency in governance could include which partner is listed as the project lead based on grant guidelines, partner weaknesses that warrant one partner to lead in a specific area and funds and resources required to support the effort of each partner.
Principle 5. Equitable resource sharing
Equitable resource sharing in community‐academic partnerships involves the identification of funds for the initial stages of project development and the establishment of subcontracts with the community. This is a commonly cited barrier across community‐academic partnerships. However, solutions have been proposed and tested within some academic institutions. Examples include having a guide for partnerships on the fiscal processes of community subcontracts, seeking supplemental funds or creating start‐up funds or cash‐limited CBOs subawards to be paid quarterly in advance at the university level. Regardless of the mechanism used to provide funding to CBOs, a description of the budget requirements and potential barriers in funding distribution should be explained to the community partner.

### 3.5 External Factors

Before relationship and partnership building, each partner should assess the external factors that could positively or negatively impact these processes. External factors include social determinants of health, structural racism, bias, resources, policies and the community demand for services. For example, each partner, especially researchers, must be aware and acknowledge their personal history, experiences or absence of experiences, along with their biases as they could impact the decision‐making for each stage of the research process. Some may be harder to address such as policies, which create power differentials in partnerships or negatively impact access to resources (i.e., an input).

#### 3.5.1 Phase I: Relationship Building

Relationship building is important and requires a minimum of one academic partner and one community partner. Partners can be added or removed as needed but retained until post‐research study. To aid in partnership development, a database of existing, past or potential partners should be created. This database should include partner mission, research interests and points of contact. During the first encounter, each partner should identify the organization's mission, vision and history along with their research interests. If they do not align, each partner can help the other identify a partner with similar research interests. If they align, the partners should discuss the research initiative and if the partnership is practical based on external and internal factors. In either case, communication should be ongoing between partners to provide updates on needs and research interests, current and future projects including those research‐related and personal matters, if appropriate. This allows each partner to see the humane side and their character, a strategy to prove one is trustworthy even when there is little to no trust in an institution. During this phase and throughout the partnership, it is important to create a safe space for all partners. It is not uncommon to initiate the needs and capacity assessments along with a root cause analysis to help determine perceived factors contributing to and potential strategies to address the public health issue.

Funds may be limited or unavailable to build relationships. However, the researcher can take advantage of community and networking events. One can also offer events or workshops to support the CBO. In addition, the researcher can follow the CBO on social media and support their efforts via likes or comments. In some instances, researchers have access to grant opportunities that support partnerships or the institution offers networking events for community organizations and researchers. There can also be periodic check‐ins via coffee breaks or lunches.

### 3.6 Inputs for Relationship and Partnership Building

Each partner should determine what they can invest in relationship and partnership building. Three months is a minimal amount of time needed to build a relationship; however, it may take longer depending on the partners' preference and status (i.e., new, existing or former). If a relationship is non‐existent and time is unavailable before a grant deadline, being honest and transparent in grant requirements, intentions and expectations is critical. Partners should engage in relationship‐building meetings while awaiting grant outcomes. Ongoing communication between community and academic partners should be established during and outside research initiatives. The amount of time to conduct the research process varies.

Knowledge and skills are transferable across partner(s). To ensure each partner has the competencies to conduct the research process equitably, they can create and/or identify training opportunities. It is important to understand that mistakes will be made throughout the process. Resources of each partner and outside the partnership (e.g., additional grants and partnerships) are identified to conduct the research. Understanding inputs can reveal potential barriers and facilitators of a potential partnership and lead to brainstorming sessions on how to address those gaps.

#### 3.6.1 Phase II: Form Partnership

A community partner can identify researchers or research institutions and vice versa for partnership development. Within community–academic partnerships, the CBO typically serves as the community partner. Other stakeholders such as industry (e.g., pharmaceutical companies), providers, government and media can be the sole or an additional partner. These partners have varying research experiences. Researchers can represent different institutions. Once formed, the researcher and CBO leads for the partnership have an in‐depth dialogue about each institution. For example, researchers should explain the policies of the academic institutions and funding agencies along with research team interests and operations. This will allow the CBO to identify if and how they can collaboratively work with the academic partner including the grant writing process. The researcher should be ready to equip the community leaders and members with the necessary knowledge and skills to be equitable partners. In addition, the researcher should advocate for the community partner(s) and their identified roles in the research process at the institutional and societal levels.

The community partner should share their CBO operations to determine how they can merge with academics to conduct research. They select an individual(s) within their organization to be involved throughout research planning, implementation and evaluation procedures and link the researcher to leadership, the community and/or patients as needed. This individual can serve as a co‐principal investigator (co‐IP) or a co‐investigator depending on the funding and academic institution. Members of the target population can be identified within or outside of the organization to serve in other research capacities (e.g., community advisory board member, consultant and advocate). A major role of the community partner and its constituents is to advocate for research partners within the community.

In some instances, engaging other stakeholders across domains including policymakers and health system decision‐makers is necessary to promote sustainability in changes deemed impactful on community health outcomes.

Although it can be challenging, it is important to establish governance, provide structure, offer balance, allow fluidity in the partnership and protect both partners to ensure its success. This should be reflected in a memorandum of understanding (MOU), which will allow a streamlined collaboration and mutual accountability. Principles of Community Engagement [12], Community‐Based Participatory Principles [11], Good Clinical Practice [45] and Belmont principles [46] should be introduced and applied moving forward. Diversity through an intersectional lens for each partner and their associated team members are needed to ensure the final programme or product is innovative and reflects the target audience's needs. Furthermore, it is important to offer different modalities (e.g., interpreter) for partners to conduct research and engage participants in the process to ensure inclusion in participation. Collectively, the application of these strategies will ensure that the community partner(s) are equitably engaged in each phase of the research process; the research process is conducted ethically; and outcomes are mutually beneficial. The assessments initiated in phase one or between phases one and two should be completed to inform the research.

### 3.7 Bidirectional Learning

Bidirectional learning is a key component of the partnership, and it is ongoing once the partnership has formed. It involves the two‐way exchange of knowledge of governance and individual practices of respective institutions, roles and responsibilities and resources and innovations of both the academic and community partners. In some instances, these partnerships can include more than one academic partner and/or community partner which can span across domains that yield cooperative learning. A common language is developed and used between both partners in the research process to co‐create a product meeting both their needs. Collectively, this promotes fairness, integrity, respect and trust within the partnerships' performance and decisions. However, bidirectional learning may not occur through every phase of the research process due to the degree of engagement chosen by both partners or institutional policies.

#### 3.7.1 Phase III: Form Research Team

The research team constitutes select members from the academic institution(s) and community‐based organization(s). Members of the community at large (e.g., CBO leaders, patients, caregivers, clients) can also be a part of the research team. Individual(s) who develop, implement and evaluate the research process should be on the team. The researcher should release the autonomy of component(s) within the research process to the community if permissible by regulatory (i.e., IRB) and legal affairs. The role (e.g., PI, co‐PI or team member) and degree of involvement of the team member(s) in each phase could vary based on the research activities and partner preference. This is how we define equitable partnerships. The goal is for bidirectional, yet equitable partner involvement throughout the research process.

#### 3.7.2 Phase IV: Conduct Research Process

We describe the systematic research process and procedures to be conducted by the research team of the equitable community–academic partnership.

##### 3.7.2.1 Stage 1: Define the Problem

To define the research problem, the research team must explore and prioritize the issues, needs and strategies of a target audience. A common way to do this is to conduct needs and capacity assessments along with a root cause analysis. Community partners can further explain factors fuelling a health issue based on their lived experiences or existing data. They can also assist the researcher in how to engage (i.e., language, culture, social norms and power structure) the target audience to conduct the assessments. This process could potentially be initiated or completed from Phase I *Relationship Building* and/or Phase II *Form Partnership.* This stage of the research is necessary to obtain data to provide a comprehensive, yet accurate representation of chronic disease health outcomes including disparities and inequities. The data, in addition to the feedback from the community partner, will aid in advancing health equity by (1) gaining a better understanding of the health problem, (2) guiding the allocation of resources, (3) prioritizing the intervention and/or (4) making health system or policy changes.

##### 3.7.2.2 Stage 2: Specify the Research Question

Specifying the research question allows both the researchers and the community members to clearly understand ‘what’ is being investigated to improve health outcomes. This question should fill a gap in the field but be equally important to the community partner. Different community contexts, including politics, society, culture, economics, environment and history, should be considered when developing the question. Therefore, researchers and community members should co‐define the research question(s) and study design using their experiences and findings from the literature, data sets and/or assessments. If needed, researchers should provide community members with the components of a research question and how they are developed. Researchers need to be open to altering the study objective and the associated research question(s) based on community partners' feedback. This will also ensure that appropriate data is being collected and will be beneficial to the community.

##### 3.7.2.3 Stage 3: Design the Research

The research design is the ‘blueprint’ for the type of research conducted and ensures it is conducted efficiently. It includes the study design, target population, intervention (if applicable), recruitment and retention strategy(ies), sample size, data collection, measures, data analysis, dissemination and compensation. It is important for the community to be involved in the design of these components. This will ensure accuracy, relevance, fairness and cultural sensitivity. Furthermore, it will promote the likelihood of representation of underrepresented research groups and equity in the methods and data. Community partners can lead and/or implement these research methods pending policies and practices of the institution and CBO. Some study components are usually led by researchers such as the study design, data analysis plan and sample size calculation. While most community partners may not understand or be interested, it is important to discuss these for input. If interested, community partners can help identify the best ways to engage them to learn and/or participate in these components' implementation. Last, the data presentation plan may vary and should include table and figure development by both partners to ensure comprehensiveness and accuracy to all stakeholders. Academic partners may need to explain certain research components (e.g., research methodologies and study designs and how to choose each, compensation) to increase the community partners' degree of participation. Guides are available to aid in the development of the study design. For example, the Urban Institute offers guidelines for establishing compensation for community engagement, in which experience, skills, time and geographic location must be considered.

After designing the research and, in some cases, obtaining grant funding, the IRB must approve before data collection if not already received. It is important to understand legal and regulatory affairs often have difficulty understanding community partners in research roles. Getting studies approved that include multiple populations, many languages and tailored materials for diverse populations can also be difficult. This can hinder the inclusion of diverse populations and partnership equity. Researchers should have discussions with legal and regulatory affairs before or immediately upon receipt of the notice of grant award to understand the ‘rules of engagement’.

##### 3.7.2.4 Stage 4: Collect the Data

The systematic process of gathering and measuring the data is known as the data collection process. This process can vary in purpose, type and length of time. The researcher, community partner or both can conduct this process. Data collection should be equitable and inclusive to promote the representation of all diverse voices representing the study's eligibility criteria. Community partners can ensure proper transcreations of data collection tools, ensure one's bias is checked when designing surveys and interview/FG guides and identify the best mode to collect the data. A research team member should train, conduct and/or observe community members to ensure data collection is occurring accurately. Periodic check‐ins should occur to ensure data collection is going as planned.

##### 3.7.2.5 Stage 5: Analyze the Data

Data analysis is one of the easier components of the research process. It can be time‐consuming, depending on the type of analysis. It involves cleaning and organizing data using descriptive statistics to describe data and testing hypotheses and models (i.e., inferential statistics), developing themes and sub‐themes (i.e., qualitative analysis) or both (i.e., mixed methods). Community members can determine if and the extent they will be involved. They should be involved in data interpretation, especially for qualitative data to reduce bias and to understand context. Results and their interpretation should be agreed upon by both partners.

##### 3.7.2.6 Stage 6: Disseminate the Results

Researchers have always disseminated study results in the academic setting including oral/poster presentations, manuscripts and performance progress reports. Community partners can inform and/or develop sections of these tangibles. They can co‐present at conferences. The provision of study results to community partners and the community at large varies for researchers across the translation continuum. However, it is an ethical responsibility to share study results with the community, highlighting information that the community can put into action. Community partners can take the lead in developing content, format and/or strategy(ies) for community distribution. The degree and strategies used for dissemination may depend on the budget, time and demand. It is important for both partners to identify and engage stakeholders across domains (e.g., policymakers) and develop deliverables (e.g., one‐pager, presentation) to disseminate findings tailored to their needs to inform decisions that could impact health equity. Researchers should go to community events and answer questions regarding findings, impact on health and the next steps for research when possible. Cunningham‐Erves et al. [47] developed the Community‐Engaged Research Dissemination Model proposing community research dissemination at three timepoints—Preliminary research phase (i.e., grant submission and results); throughout the research process (i.e., update on study progress and preliminary results); and post‐study (i.e., results, implications and next steps). Based on the partners' preference, the partnership could end post‐research dissemination or be sustained to extend research.

### 3.8 Outcomes

There are short‐term, intermediate and long‐term partnership outcomes. Short‐term outcomes include knowledge of the research process and community roles, attitudes, partnership equity, shared language, transfer of skills between partners and trust or trustworthiness in the partner and research process. The 10 principles of trustworthiness can be used to guide these partnerships [48]. The intermediate outcome is policy change(s), shared decision‐making and research implementation outcomes. The long‐term outcome is partnership sustainability and return on investment in economics, social context and environment. Collectively, the partners can identify additional outcomes for exploration as a result of their partnership. Furthermore, the outcomes can be used to sustain and foster new relationships to engage bidirectional equitable community–academic partnerships.

## 4 Discussion

Establishing a framework to engage in equitable, bidirectional community–academic partnerships to conduct collaborative research is necessary to advance health and improve outcomes. We describe the development of the BEE research framework for community and academicians seeking to engage in a research process that is equitable yet bidirectional. Key framework components demonstrate that equity is defined by the degree of involvement in each phase of the research process as defined by each partner, and bidirectional learning is ongoing with educational opportunities identified by both partners. This framework is distinct from past frameworks, models and principles related to CBPR by proposing four phases of research that are shaped through the intersection of equity, CE [12], which includes CBPR [11] and partnership principles [49]. The framework could standardize the research process's planning, implementation and evaluation between community and academic partners.

Engaging researchers and community members was critical in the development of the framework. Past studies demonstrate an increased likelihood of programme acceptance and improved outcomes when stakeholders, especially community leaders and members, are engaged in the planning phases of research [12, 37, 46]. Academic researchers and community leaders and members found the initial framework acceptable during the original implementation process [50] and perceived that it represented an ideal process for conducting research that was bidirectional and equitable, suggesting further revisions of this model with multilevel stakeholder input should further improve the degree of acceptability. We further offer competencies and tools that can be used by both academic and community partners to determine the readiness and capacity to conduct the work proposed in this framework. The establishment of competencies can reveal training needs, ensure appropriate training opportunities are chosen and inform metrics to determine the degree of equity and engagement in the partnership. Long‐term application of this framework can yield a bank of evidence‐based strategies and tools.

### 4.1 Strengths and Limitations

A major strength is the intersection of equity, CEnR inclusive of CBPR, partnership principles and stakeholder feedback used to inform framework development. We offer strategies, tools and competencies that academic and community partners need to conduct this process. However, there are weaknesses. Real‐life application of the framework could be difficult depending on the knowledge, skills, resource and time required of each partner to implement the framework. Only three stakeholder perspectives, researchers and community leaders and members who were primarily African American, informed the development of this framework, which may limit the framework's scope. However, this model provides the foundation to conduct this process and is scalable to other minoritized groups. Future studies should confirm these results with the revised framework.

## 5 Conclusion

We developed the BEE research framework using a narrative literature review, including models, frameworks and measures to determine partnership equity and synergy along with applying existing CEnR and CBPR principles. We also had a review by scientific experts, community members and CBO leaders to design the framework further. This framework adds to the literature by offering a standardized, stepwise approach guided by stakeholders to engage community and academic partners in the research process equitably. External factors that influence community–academic partnerships, inputs needed to engage in the research process and areas to evaluate the community–academic partnership have been provided to explain and measure bidirectional, equitable engagement in these partnerships. We further offer competencies and tools for each partner, which is often lacking with other frameworks. Using equitable, bidirectional engagement processes while community–academic partnerships conduct research can potentially improve bidirectional learning, shared governance and ultimately partnership sustainability and research implementation outcomes. In the long term, this can lead to changes that improve health outcomes and inequities.

## Author Contributions

**Jennifer Cunningham‐Erves:** conceptualization, supervision, investigation, methodology, validation, writing–original draft, writing–review and editing, visualization, funding acquisition, software, formal analysis, project administration, resources, data curation. **Tilicia Mayo‐Gamble:** data curation, writing–review and editing, writing–original draft, visualization. **LaNese Campbell:** conceptualization, investigation, funding acquisition, data curation, formal analysis, project administration, supervision, writing–review and editing, writing–original draft, validation, methodology, visualization, resources. **Bishop Calvin Barlow:** conceptualization, investigation, funding acquisition, writing–review and editing, methodology, validation, resources, supervision, project administration. **Claudia Barajas:** funding acquisition, writing–review and editing, validation, data curation. **Jessica L. Jones:** data curation, project administration, writing–review and editing. **Karen Winkfield:** writing–review and editing.

## Ethics Statement

This study was performed in line with the principles of the Declaration of Helsinki. Approval was granted by the Meharry Medical College Institutional Review Board (Protocol #: 23‐05‐1305).

## Consent

All participants provided electronic consent via REDCap before participating in the study.

## Conflicts of Interest

Dr. Jennifer Cunningham‐Erves is an education consultant and advisory panel member for MERCK. She is also an education consultant for the Foundation for Sarcoidosis Research. These companies had no role in the development, writing or decision to publish this article. The other authors declare no conflicts of interest.

## Supporting information

Supporting information.

Supporting information.

## Data Availability

Due to confidentiality agreements, supporting data cannot be made openly available.
