# Erratum to: “The Bidirectional Engagement and Equity (BEE) Research Framework to Guide Community‐Academic Partnerships: Developed From a Narrative Review and Diverse Stakeholder Perspectives”

**DOI:** 10.1111/hex.70031

**Published:** 2024-09-10

J. Cunningham‐Erves, T. Mayo‐Gamble, L. Campbell, et al., “The Bidirectional Engagement and Equity (BEE) Research Framework to Guide Community‐Academic Partnerships: Developed From a Narrative Review and Diverse Stakeholder Perspectives,” *Health Expectations* 27, no. 4 (August 2024): e14161, https://doi.org/10.1111/hex.14161.

In Figure 2, “The bidirectional equity and engagement (BEE) research framework”, the text “research teams” and “academic partners” were placed incorrectly. The text should have read: “Academic partner” linked with community partner, and “research teams” in the second level linked with community members, patients, advocates, and caregivers.


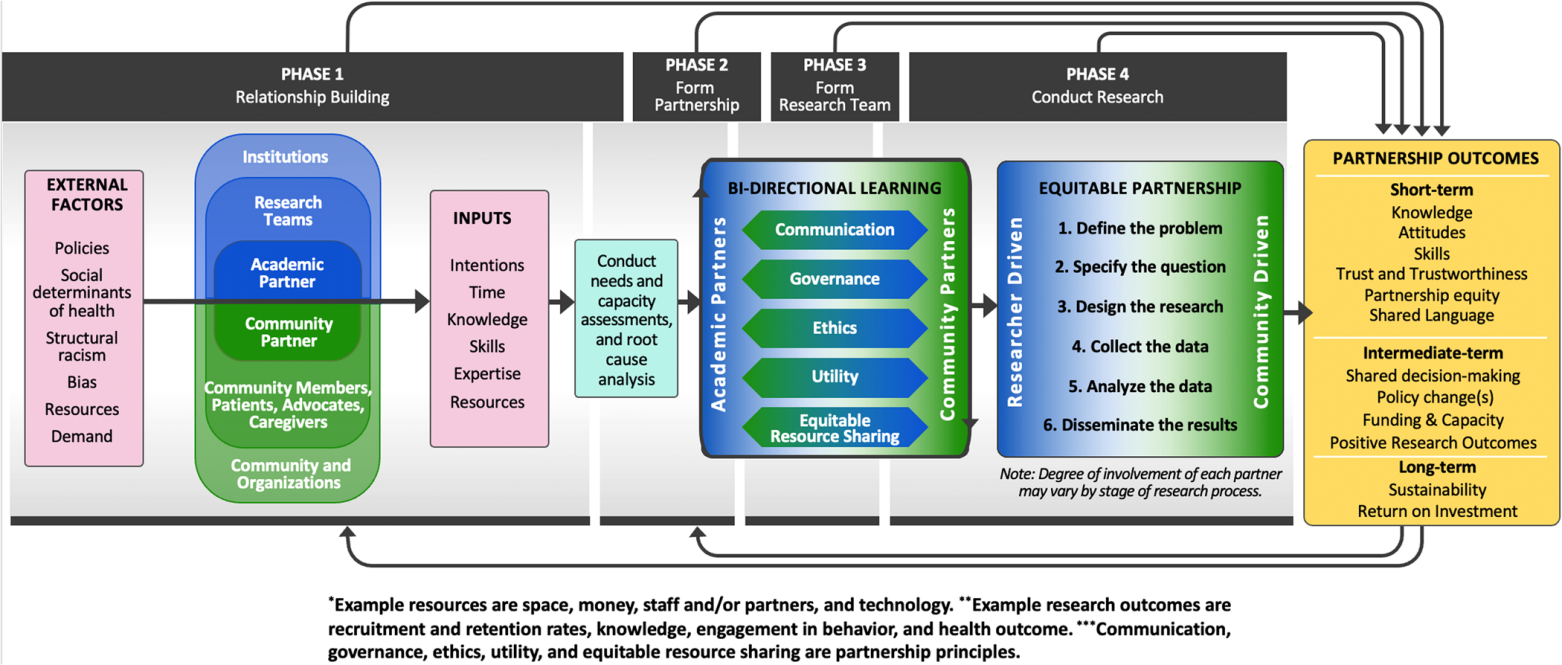


We apologize for this error.

