# Supplementary figures and images for: The Bidirectional Engagement and Equity (BEE) Research Framework to Guide Community–Academic Partnerships: Developed From a Narrative Review and Diverse Stakeholder Perspectives
Source: Health Expect. 2024 Aug 1;27(4):e14161. doi: 10.1111/hex.14161 (PMC11292665; doi:10.1111/hex.14161)

**Supplemental File 1**

Original Framework


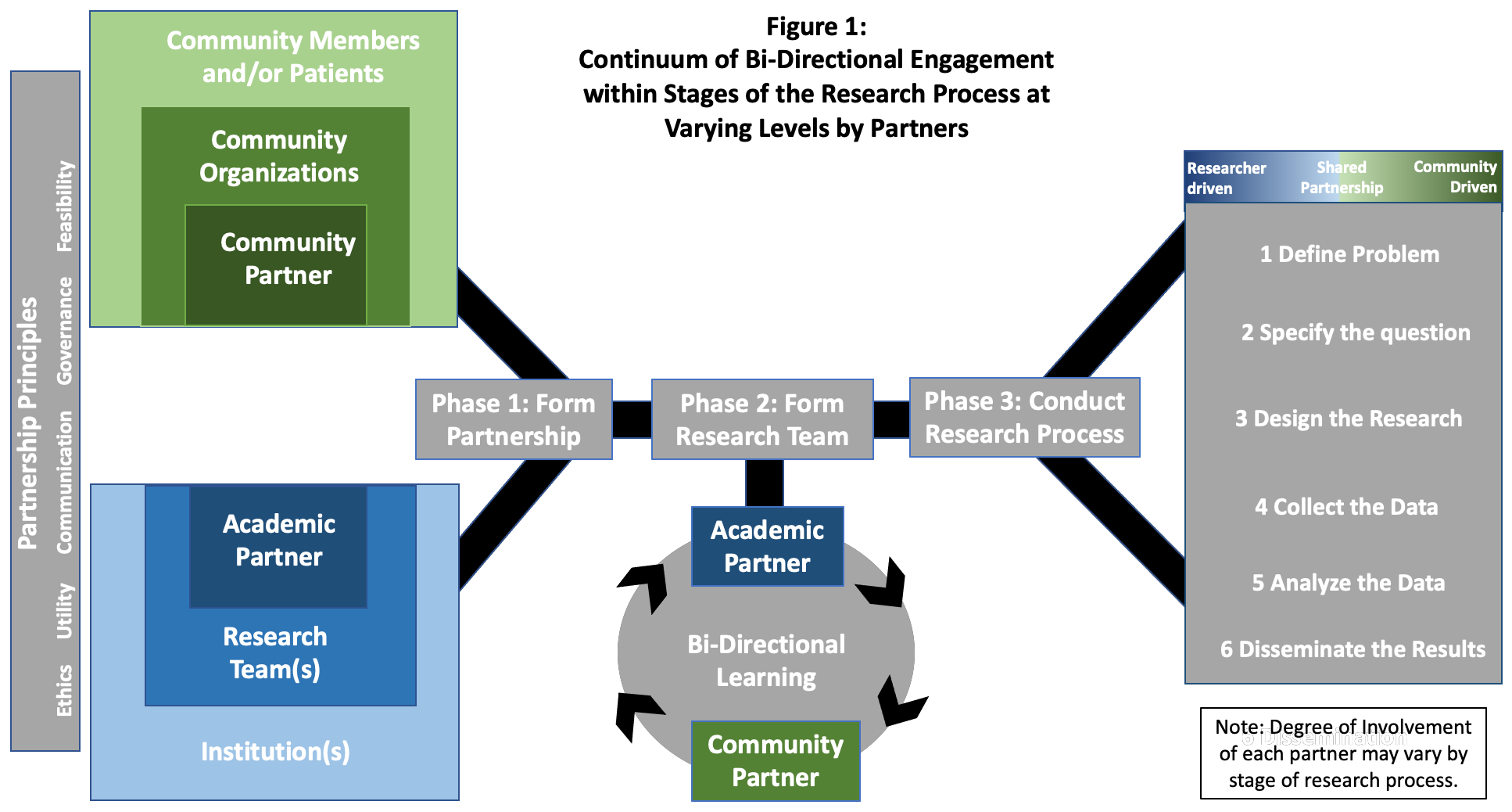

Supplement: Supplementary file 1 — Supporting information. [file HEX-27-e14161-s002.docx]
