# Supplementary material for: The Bidirectional Engagement and Equity (BEE) Research Framework to Guide Community–Academic Partnerships: Developed From a Narrative Review and Diverse Stakeholder Perspectives
Source: Health Expect. 2024 Aug 1;27(4):e14161. doi: 10.1111/hex.14161 (PMC11292665; doi:10.1111/hex.14161)
Supplement: Supplementary file 2 — Supporting information. [file HEX-27-e14161-s001.docx]

**Supplemental File 2. Narrative review of the models or frameworks along with principles related to bi-directional, equitable partnerships**

**Methods**

The narrative review was conducted in three electronic databases *PscyINFO*, *PubMed*, and *CINAHL* from November 2020 to July 2022. We developed a search criterion using key terms ‘framework’, ‘model’, ‘community-academic partnerships’, ‘bi-directional learning’, ‘health equity’, and ‘community-based participatory research (CBPR)’ across the databases. These search teams were centered around three concepts: frameworks and models related to community-academic partnerships, partnership and equity principles, and health equity. The term “CBPR” complicated the search as many articles discussed research results or the engagement process.

**Inclusion criteria**. Inclusion criteria were articles describing frameworks or models that: 1) describe the bidirectional relationship and function of community-academic partnerships; or 2) promote health equity. We also viewed articles which discussed or described the application of partnership principles, interventions that promote bidirectional community-academic partnership principles or health equity and provided the recommendations or guiding principles for community-academic partnerships to conduct the research process. There were no restrictions on the target population.

**Screening procedures**. All citations with full text availability or requested access were combined into one database and duplicates removed. When no new characteristics related to community-academic partnerships and the conduct of the research process emerged in the literature, we reached saturation and completed the search. Two research team members reviewed and categorized the articles. Then, they compared their classification and discussed until a consensus was reached per article to yield a final set of articles.

**Data extraction.** This search yielded 15 articles to be analyzed for content related to bidirectional and/or equitable community-academic partnerships. Content of models and framework related to community-academic partnerships and the conduct of the research process were scanned by the two research team members. This search also yielded 24 interventions, guiding principles, and/or recommendations on how to conduct a bi-directional, equitable community-academic partnership. This data was placed in a table and reviewed by the team members. These results were categorized according to inclusion criteria by the two team members and modifications were made after their discussion.

**Figure 1: Prisma Flow Diagram for Narrative Literature Search**

**Identification of studies via databases and registers**

Records removed *before screening*:

Duplicate records removed (n = 215)

Records marked as ineligible by automation tools (n = 12,523)

Records marked as ineligible by human review (n = 649)

Records removed for other reasons (n = 4807)

Records identified from*:

PubMed (n = 9,784)

CINAHL (n = 17,039)

PsycInfo (n = 2633)

**Identification**

Records excluded**

(n = 11,165)

Records screened

(n = 11,262)

Reports not retrieved

(n = 0)

Reports sought for retrieval

(n = 97)

**Screening**

Reports excluded:

Reason 1 (n = did not describe a model/framework)

Reason 2 (n = absence of a community-academic partnership)

Reason 3 (n = did not support guiding principles)

Reports assessed for eligibility

(n = 97)

Studies included in review

(n = 15)

**Included**

*Consider, if feasible to do so, reporting the number of records identified from each database or register searched (rather than the total number across all databases/registers).

**If automation tools were used, indicate how many records were excluded by a human and how many were excluded by automation tools.

**Results.** Figure 1 yields the narrative review process. The citation details of 29,456 articles (PubMed=9,784; CINAHL=17,039; and PsycInfo=2633) were reviewed by two research team members. Two hundred fifteen duplicates were removed, leaving 29,241 articles to be reviewed further. After careful review or the titles and abstracts the non-duplicative records, 15 articles focused on bidirectional and/or equitable community-academic partnerships (Table 1). We also identified 24 research articles to inform key community-academic partnership principles. Examples include Mullins et al. [1], Marrone, Nieman, and Coco [2], and Hohl, Neuhouser, and Thompson [3].

| **Table 1: Literature Review Results of the 15 Frameworks to Inform the *Bi*-Directional *E*ngagement and *E*quity (BEE) Research Framework** | | | |
| --- | --- | --- | --- |
| **Author, Year** | **Framework**  **Study Title** | **Model Purpose** | **Constructs (C), Domains (D), Criteria (Cr) Principles (P) or Values (V)** |
| Michie et al., 2011 (4) | The behaviour change wheel: A new method for characterizing and designing behaviour change interventions | -Provides a systematic method to understand the nature of behavior, and an appropriate system for characterizing interventions and their components to make use of this understanding. | 1. Capability (C)  2. Opportunity (C)  3. Motivation (C) |
| Dover & Belon, 2019 (5) | The health equity measurement framework: a comprehensive model to measure social inequities in health | Measures direct and indirect effects of social determinants on health (in)equity. | 1. Socio-economic, cultural, and political context (C) 2. Social stratification process (C) 3. Social location (C) 4. Material circumstances (C) 5. Social circumstances (C) 6. Biology (C) 7. Environment (C) 8. Health-related Behaviours (C) 9. Health Beliefs (C) 10. Pre-existing health state (C) 11. Psychosocial stressors (C) 12. Appraisal and coping (C) 13. Stress response (C) 14. Health state (C) 15. Need (C) 16. Health policy context (C) 17. Availability of health-promoting resources (C) 18. Acceptability, appropriateness, safety, effectiveness, and continuity (C) 19. Accessibility (C) 20. Utilisation of health-promoting resources (C) 21. Outcomes (C) |
| Birken et al., 2017 (6) | Combined use of the Consolidated Framework for Implementation Research (CFIR) and the Theoretical Domains Framework (TDF): a systematic review | CFIR guides systematic assessment of multilevel implementation contexts to identify factors that might influence intervention implementation and effectiveness. TDF provides a framework to help apply theoretical approaches to interventions aimed at behavior change. | CFIR Domains  1.Innovation (D)  2.Outer Setting (D)  3.Inner Setting (D)  4.Individuals (D)  5.Implementation Process (D)  TDF Domains  1.Knowledge  2.Skills  3.Social/Professional role and identity  4.Beliefs about capabilities  5. Optimism  6. Beliefs about consequences  7. Reinforcement  8.Intentions  9.Goals  10.Memory, attention, and decision processes  11.Environment context and resources  12. Social influences  13. Emotion  14. Behavioral regulation |
| Barker et al., 2021 (7) | Community-engaged healthcare model for currently under-served individuals involved in the healthcare system | -Promotes a community-engaged healthcare | 1. Client and healthcare systems: the ecological model (C)  2. Rising agency and shifting power: Barriers and enablers (C)  3. Culture shifting (C) |
| Farley-Ripple et al., 2018 (8) | Rethinking Connections Between Research and Practice in Education: A Conceptual Framework | -Promotes bidirectional conversation on an issue and guiding inquiry linking research and practice. | 1. Gaps in Assumptions & Perspectives (D)  2. Depth of Use (D)  3. Depth of Production (D) |
| Sanchez et al., 2021 (9) | CBPR Implementation Framework for Community-Academic Partnerships | Promotes intervention uptake systematically and intentionally in partnering and values of equity. | 1. External and internal contexts (D)  2. Structural and relational elements of partnering processes (D)  3. Intervention and research (D) |
| Corbie-Smith et al., 2018 (10) | Stakeholder-driven, consensus development methods to design an ethical framework and guidelines for engaged research | -Promote a review and conduct of scholarship with engagement (i.e., research). | 1. Vision of equitable and just research (D)  2. Relationship dynamics (D)  3. Community-informed risk/benefits assessment (D)  4. Accountability (D) |
| Calancie et al., 2021 (11) | Consolidated Framework for Collaboration Research derived from a systematic review of theories, models, frameworks and principles for cross-sector collaboration | Seeks to promote cross-sector collaborations to guide research and practice. | 1. Community context (D)  2. Group composition (D)  3. Structure and internal processes (D)  4. Group dynamics (D)  5. Social capital (D)  6. Activities that influence or take place within the collaboration (D)  7. Activities that influence or take place within the broader community (D)  8. Activities that influence or take place both in the collaboration and in the community (D) |
| Ahmed and Palermo, 2010 (12) | Community Engagement (CE) in Research: Frameworks (F) for Education and Peer Review | N/A | N/A |
|  | F1: NIH Director’s Council of Public Representatives’ Framework for Education on Community | Operationalizes community engagement values and potential outcomes of each value. | 1. Definition and scope of community engagement in research (P)  2. Strong community-academic partnership (P)  3. Equitable power and responsibility (P)  4. Capacity building (P)  5. Effective dissemination plan (P) |
|  | F2: Framework for Peer Review on CE | Provides criteria for the review of proposals involving CE | 1. Peer reviewers understand and have experience conducting research that involves CE (Cr)  2. Peer reviewers understand the value added by public review panel members (Cr)  3. The application provides evidence of an equitable partnership between the investigators and the community partner (Cr)  4. In the application, the investigators have defined the relevant community or communities (Cr)  5. In the application, the academic coinvestigators have identified the appropriate community or communities for the project, and the community coinvestigator has identified appropriate research partner for the project (Cr)  6. CE is an integral part of the research described in the application (Cr)  7. The community played an appropriate and meaningful role in developing the application (Cr)  8. The application calls for an appropriate division of funding among partners (Cr)  9. The research project described in the application is based on sound science (Cr)  10. The project described in the application includes training opportunities (Cr)  11. The project described in the application will be conducted in an appropriate environment (Cr)  12. The project described in the application will have a measurable impact (Cr) |
| Reese et al., 2019 (13) | The Development of a Collaborative Self-Evaluation Process for Community-Based Participatory Research Partnerships Using the Community-Based Participatory Research Conceptual Model and Other Adaptable Tools | Promotes the use of culture-centered approach in the context of CBPR-based health education research. | 1. Internal and External Contexts (D) 2. Partnership Processes (D) 3. Intervention and Research (D) 4. Outcomes (D) |
| Ward et al., 2018 (14) | A conceptual framework for evaluating health equity promotion within community-based participatory research partnerships | Offers a synergistic framework that integrates community and academic contributions to evaluate partnership effectiveness in addressing health inequities. | 1. Structural characteristics 2. Group dynamics characteristics of effective and equitable partnerships 3. Environmental characteristics 4. Partnership programs and interventions |
| Baquet, 2012 (15) | A model for bidirectional community-academic engagement (CAE): overview of partnered research, capacity enhancement, systems transformation, and public trust in research. (Community-Academic Model) | Offers a framework that promotes long-lasting, bidirectional partnerships between community and academia | 1. Administrative infrastructure (D) 2. Regional partners and offices (D)   3. Partnerships (D)  4. Leveraging resources for sustainability (D)  5. Multidisciplinary Research (D)  6. Science-guided policy, policy research, and community advocacy (D)  7. Diversity in clinical trials participation (D)  8. Program evaluation and research (D) |
| Organizing Committee for Assessing Meaningful Community Engagement in Health & Health Care Programs & Policies, 2022 (16) | Assessing Meaningful Community Engagement: A Conceptual Model to Advance Health Equity through Transformed Systems for Health  (Assessing Community Engagement (ACE) Conceptual Model) | Promotes health equity through transformed systems for health through use of core principles and taxonomy to implement and assess CE principles. | 1. Strengthened partnerships and alliances (D) 2. Expanded knowledge (D) 3. Improved health and health care programs and policies (D) 4. Thriving communities (D) |
| Cargo and Mercer, 2012 (17) | The Value and Challenges of Participatory Research: Strengthening Is Practice  (An Integrative Practice Framework for Participatory Research) | Provides a structured process for academic and non-academic partners to develop and sustain partnerships to design, conduct, and evaluate participatory research efforts. | 1. What are the values or drivers behind the research? (D)  2. Who should participate in the research, and how do they evolve? (D)  3. How are partnerships initiated, and how do they evolve? (D)  4. What are the core elements of PR? (D)  5. What is the added value of PR in each of the research phases? (D) |
| Andrews et al., 2012 (18) | Partnership readiness for community-based participatory research | Assesses and leverages academic and community partner’s readiness to conduct CBPR. | 1. Goodness of fit (D)  2. Capacity (D)  3. Operations (D) |

**References**

**References**

1. Mullins CD, Tanveer S, Graham G, Baquet CR. Advancing community-engaged research: increasing trustworthiness within community-academic partnerships. J Comp Eff Res. 2020;9(11):751-3.

2. Marrone NL, Nieman CL, Coco L. Community-Based Participatory Research and Human-Centered Design Principles to Advance Hearing Health Equity. Ear Hear. 2022;43(Suppl 1):33s-44s.

3. Hohl SD, Neuhouser ML, Thompson B. Re-orienting transdisciplinary research and community-based participatory research for health equity. J Clin Transl Sci. 2022;6(1):e22.

4. Michie S, van Stralen MM, West R. The behaviour change wheel: a new method for characterising and designing behaviour change interventions. Implement Sci. 2011;6:42.

5. Dover DC, Belon AP. The health equity measurement framework: a comprehensive model to measure social inequities in health. Int J Equity Health. 2019;18(1):36.

6. Birken SA, Powell BJ, Presseau J, Kirk MA, Lorencatto F, Gould NJ, et al. Combined use of the Consolidated Framework for Implementation Research (CFIR) and the Theoretical Domains Framework (TDF): a systematic review. Implement Sci. 2017;12(1):2.

7. Barker SL, Maguire N, Gearing RE, Cheung M, Price D, Narendorf SC, et al. Community-engaged healthcare model for currently under-served individuals involved in the healthcare system. SSM Popul Health. 2021;15:100905.

8. Farley-Ripple E, May H, Karpyn A, Tilley K, McDonough K. Rethinking Connections Between Research and Practice in Education: A Conceptual Framework. Educational Researcher. 2018;47(4):235-45.

9. Sánchez V, Sanchez-Youngman S, Dickson E, Burgess E, Haozous E, Trickett E, et al. CBPR Implementation Framework for Community-Academic Partnerships. Am J Community Psychol. 2021;67(3-4):284-96.

10. Corbie-Smith G, Wynn M, Richmond A, Rennie S, Green M, Hoover SM, et al. Stakeholder-driven, consensus development methods to design an ethical framework and guidelines for engaged research. PLoS One. 2018;13(6):e0199451.

11. Calancie L, Frerichs L, Davis MM, Sullivan E, White AM, Cilenti D, et al. Consolidated Framework for Collaboration Research derived from a systematic review of theories, models, frameworks and principles for cross-sector collaboration. PLoS One. 2021;16(1):e0244501.

12. Ahmed SM, Palermo A-GS. Community Engagement in Research: Frameworks for Education and Peer Review. American Journal of Public Health. 2010;100(8):1380-7.

13. Reese AL, Hanza MM, Abbenyi A, Formea C, Meiers SJ, Nigon JA, et al. The Development of a Collaborative Self-Evaluation Process for Community-Based Participatory Research Partnerships Using the Community-Based Participatory Research Conceptual Model and Other Adaptable Tools. Prog Community Health Partnersh. 2019;13(3):225-35.

14. Ward M, Schulz AJ, Israel BA, Rice K, Martenies SE, Markarian E. A conceptual framework for evaluating health equity promotion within community-based participatory research partnerships. Evaluation and Program Planning. 2018;70:25-34.

15. Baquet CR. A model for bidirectional community-academic engagement (CAE): overview of partnered research, capacity enhancement, systems transformation, and public trust in research. J Health Care Poor Underserved. 2012;23(4):1806-24.

16. Organizing Committee for Assessing Meaninful Community Engagement in Health & Health Care Programs & Policies. Assessing meaningful community engagement: A conceptual model to advance health equity through transformed systems for health. NAM Perspectives. 2022.

17. Cargo M, Mercer SL. The Value and Challenges of Participatory Research: Strengthening Its Practice. Annual Review of Public Health. 2008;29(1):325-50.

18. Andrews JO, Newman SD, Meadows O, Cox MJ, Bunting S. Partnership readiness for community-based participatory research. Health Education Research. 2012;27(4):555-71.
